# Efficiency of Clinical Decision Support Systems Improves with Experience

**DOI:** 10.1007/s10916-015-0423-z

**Published:** 2016-01-20

**Authors:** Michiel C. Meulendijk, Marco R. Spruit, Floor Willeboordse, Mattijs E. Numans, Sjaak Brinkkemper, Wilma Knol, Paul A. F. Jansen, Marjan Askari

**Affiliations:** Department of Information and Computing Sciences, Utrecht University, Princetonplein 5, 3584 CC Utrecht, The Netherlands; Department of Geriatric Medicine and Expertise Centre Pharmacotherapy in Old Persons, University Medical Center, Heidelberglaan 100, 3584 CX Utrecht, The Netherlands; Department of Public Health and Primary Care, LUMC, Albinusdreef 2, 2333 ZA Leiden, The Netherlands; Department General Practice, VUmc, Van der Boechorstraat 7, 1081 BT Amsterdam, The Netherlands; Spru IT B.V., Livarstraat 57, 3573 SB Utrecht, The Netherlands

**Keywords:** Clinical decision support, Medication review, Efficiency, Usability

## Abstract

Efficiency, or the resources spent while performing a specific task, is widely regarded as one the determinants of usability. In this study, the authors hypothesize that having a group of users perform a similar task over a prolonged period of time will lead to improvements in efficiency of that task. This study was performed in the domain of decision-supported medication reviews. Data was gathered during a randomized controlled trial. Three expert teams consisting of an independent physician and an independent pharmacist conducted 150 computerized medication reviews on patients in 13 general practices located in Amsterdam, the Netherlands. Results were analyzed with a linear mixed model. A fixed effects test on the linear mixed model showed a significant difference in the time required to conduct medication reviews over time; F(31.145) = 14.043, *p* < .001. The average time in minutes required to conduct medication reviews up to the first quartile was M = 20.42 (SD = 9.00), while the time from the third quartile up was M = 9.81 (SD = 6.13). This leads the authors to conclude that the amount of time users needed to perform similar tasks decreased significantly as they gained experience over time.

## Introduction

Efficiency, or the resources spent while performing a specific task, is widely regarded as one the determinants of usability [1]. The experience users have with software has been shown to influence their attitudes towards prolonged use of it [2, 3]. However, only limited longitudinal research measuring the effects of prolonged software use on efficiency has been conducted. Some studies, in this regard, have indicated that determinants common in conventional usability research differ for experienced users [4, 5].

This lack of longitudinal research on efficiency needs to be addressed. In this study, this research interest is tested in the domain of decision-supported medication reviews. The authors hypothesize that having a group of users perform a similar task over a prolonged period of time will lead to improvements in the efficiency of that task.

## Background

### Efficiency in usability

Efficiency is being regarded as one of the main aspects determining software applications’ usability; it has been included in all major definitions of the concept [1, 6]. Users gain experience through prolonged exposure to a system. This experience has been shown to be a major determinant on their attitudes towards accepting technology [2, 3, 7, 8].

The commonly used definition proposed by the International Standards Organization (ISO) defines usability as ‘the extent to which a product can be used by specified users to achieve specified goals with effectiveness, efficiency, and satisfaction in a specified context of use’ [1]. In this context, efficiency exists of ‘resources expended in relation to the accuracy and completeness with which users achieve goals’. Theoretically the notion of efficiency can encompass any kind of resource, including money or knowledge, but in practice the concept is usually limited to indicate the amount of time spent on a certain task.

Most usability studies use experimental methods to determine systems’ effectiveness, efficiency, and satisfaction. The effect of temporality, or the degree to which these factors change over time, is largely overlooked [5]. At the same time, however, aspects dependent on temporality are accepted as major determinants: a common definition proposed by Nielsen emphasizes the importance of learnability and memorability of user interfaces [6]. Learnability encompasses the ease with which users can accomplish basic tasks when they first encounter a design, while memorability concerns the degree to which they can reestablish proficiency when reusing it. Thus, testing of usability through experiments often entails nothing but testing of learnability, measuring factors of novice rather than experienced users [4]. In experimental studies, in which participants are unfamiliar with the method or the user interface, a drop in efficiency can reasonably be expected [9].

### Efficiency in clinical decision support systems

In literature, there is consensus that usability has a significant impact on users’ adoption of clinical decision support systems [10]. However, many clinical decision support systems have not been shown to improve efficiency. While systems’ improvements in effectiveness are well-documented, studies on efficiency are lacking. A recent systematic literature review found that “contamination of clinicians in the control group, […] evaluation periods that were too brief to demonstrate an effect on efficiency, and small clinician sample sizes” made it impossible to generalize on efficiency consequences [11].

A study synthesizing features of proven effective clinical decision support systems found mixed results regarding efficiency: while embedding systems in physicians’ workflows was associated with improved effectiveness, advice presented within computerized physician order entry (CPOE) systems had negative correlations regarding success [12]. The authors explained this apparent anomaly by subscribing to the oft-mentioned ‘alert fatigue’ found in clinical decision support systems. In many systems, users are presented with a multitude of warnings and suggestions, forcing them to ignore the majority [13, 14].

## Objective

Following the above-mentioned considerations, the authors hypothesize that users grow more proficient performing similar tasks over time. The effects of experience on applying the application effectively and memorizing its functionality are assumed to lead to gradual improvements in efficiency.

This objective is investigated in the domain of clinical decision support systems, which is explained in more detail in the adjacent section. The research question reads: Does the time physicians and pharmacists use to systematically optimize the medical records of polypharmacy patients decrease as they gain experience?

A secondary interest is how users allocate their time during the task. This information can be used to improve decision support systems’ workflow and user interface.

### Domain: Decision-supported medication reviews

The chronic use of multiple medications, while often indicated in older patients, has been shown to lead to adverse drug reactions. A comprehensive Dutch study showed that 5.6% of Dutch hospital admissions are due to medication-related problems [15]. Appropriate prescribing in older people is challenging, because of age-related changes in pharmacokinetics and pharmacodynamics, multimorbidity and polypharmacy [16, 17].

Clinical medication reviews are oft-mentioned approaches to improve this situation. Clinical medication reviews are ‘structured, critical examination[s] of the patient’s medicines with the objective of reaching an agreement with the patient about treatment, optimizing the impact of medicines, and minimizing the number of drug related problems’.

Several clinical medication review methods have been developed to aid physicians and pharmacists in optimizing their prescriptions for these polypharmacy patients [18, 19]. Implementations of these methods as decision support systems have been proven to improve the quality of prescriptions in research settings [9, 19].

A major disadvantage of these methods, however, is that physicians need more time to perform them than they do to perform their usual care methods. In a 2009 study in which the Polypharmacy Optimization Method (POM) was tested, Drenth-van Maanen et al. found that performing a medication review with the structured method took more time (16.7 min) than performing one without (8 min) [19]. A more recent study, in which a decision support system facilitating the Systematic Tool to Reduce Inappropriate Prescribing (STRIP) was validated, Meulendijk et al. found that the average time physicians needed to optimize a patient’s health record with the STRIP method was 24 min, while the time needed without any structured approach was 13 min [9].

In this domain, the efficiency of any structured method is of major importance. So far, research has shown that effectiveness in this domain can improve with structured methods, but longitudinal research reporting on their efficiency is lacking.

## Method

### Setting

The data was collected within the intervention arm of a randomized controlled trial [20]. This trial aimed at testing whether or not the use of clinical medication reviews can reduce inappropriate drug use of older people. The randomized controlled trial included 500 patients from 25 practices in Amsterdam, the Netherlands. They were eligible for inclusion if they were 65 years or older, suffered from pre-specified geriatric symptoms in general practice, and chronically used at least one prescribed drug. The primary outcomes were quality of life and presence of geriatric symptoms, with secondary outcomes focusing on costs, feasibility, number of drug-related problems, adherence, and satisfaction. The results have not been published yet. A more detailed description of the trial’s design and intended outcomes can be found in Willeboordse et al. [20].

Patients of 13 intervention practices received clinical medication reviews, while those in the other practices served as a control group. The data for this study was collected from the clinical decision support system that was used to perform the intervention.

### Operationalization

In an attempt to answer the research question, the concept of efficiency was operationalized for the domain of decision-supported medication reviews. Teams existing of independent physicians and pharmacists form the users, whose goal it is to conduct clinical medication reviews. To account for efficiency’s definition’s concepts of accuracy and completeness, users were required to have responded to all pieces of advice, and to have assessed all aspects of the structured medication review. The expended resource was operationalized as time. Table 1 summarizes the operationalization of efficiency for this domain.

**Table 1:** Operationalization of the concept of efficiency in the domain of structured medication reviews

Efficiency	Operationalization
*resources* expended	Time (in seconds)
in relation to the *accuracy*	Having responded to all pieces of generated advice
and *completeness*	Having finished all steps of the STRIP medication review method
with which *users*	Teams of one independent physician and one independent pharmacist
achieve *goals*	Medication reviews

### Participants

For the randomized controlled trial, five expert teams, each consisting of an independent physician and an independent pharmacist, were recruited through a convenience sample. As one team had a variable composition, and another team performed only five medication reviews, three teams’ data were used. Of these three teams, all participants were female. Two of the physicians were general practitioners; one was a nursing home physician.

### Procedure

The participating teams were tasked with conducting structured medication reviews for fifty patients included in the intervention arm of the trial. This assessment was carried out according to the STRIP, which is the preferred method according to the Dutch multidisciplinary guideline on polypharmacy in elderly patients [21]. In the trial, it was facilitated by the clinical decision support system described in the next section. The medication reviews allowed the teams to identify underprescribing, overtreatment, clinical interactions, contra-indications, and inappropriate dosages in patients’ health records. They were then able to adjust the health records by prescribing new medications, removing superfluous ones, or altering dosages.

### Instrument

The clinical decision support system that was used to answer the research question is the STRIP Assistant (STRIPA), a web application that facilitates the structured pharmacotherapeutical analysis of the STRIP method [22]. STRIPA displays a single patient’s health record at a time, showing his or her diseases, medications, symptoms, and relevant measurements or observations. Users are guided through the analysis steps to optimize a patient’s medication; first, they manually assign drugs to the diseases for which they have been prescribed; second, they add any missing medication for which there is an indication; third, they eliminate superfluous ineffective drugs or drugs for which there is no appropriate indication; fourth, they check for any relevant clinical interactions between drugs; finally, they readjust dosages if necessary. In all these steps, except the first one, STRIPA generates advice based on (inter)national guidelines, most notably the START/STOPP criteria and guidelines for medication safety recommended by the Royal Dutch Pharmacists Association [23, 24]. The pieces of advice arrived at through these guidelines are used to alert users of anomalies and suggest appropriate actions in a non-intrusive fashion. A more detailed description of the software’s architecture, design, and functioning is described in Meulendijk et al. [22]. A video demonstrating the use of the STRIP Assistant can be viewed here: http://videodemo.stripa.eu/english/ [25].

### Outcome measure

The main outcome measure was the time the participating teams required to conduct a medication review. Of interest was the difference in time between teams’ earlier reviews and their later ones. Users’ actions were automatically registered in logs. Not only the actions they performed were recorded, but also how long they hesitated in between them. Logs consisted of a timestamp, a description of the action taken, the analysis phase in which it was taken, and a list of associated objects, such as medications or diseases. Table 2 shows an example of a log.

**Table 2:** A sample log showing the addition of a medication (lactulose) to a disease (obstipation) as part of an underprescribing intervention

Log ID	Value
Action description:	addedObj[ect]
User interface phase:	Underprescribing
Timestamp:	November 1st, 2013, 15:00:01
Associated objects:	1. Medication: lactulose 667 mg/ml, apply once daily. 2. Disease: chronic obstipation

### Analysis

Clickstream analysis was used to gain insight into the recorded logs of users’ actions. A clickstream is an “electronic record of a user’s activity on the Internet” and is an often used method for gaining insight into people’s behavior with web applications [26, 27].

For the statistical analysis, a linear mixed model with fixed effects was adopted. In this model, teams served as subjects, with repeated measurements being the medication reviews they performed. To account for changes in difficulty of health records, the numbers of patients’ diseases and medications were incorporated into the model as covariates. Additionally, to account for the differences in the number of advices, the number of recommendations generated by the system per review was incorporated as a covariate.

## Results

### Descriptive statistics

The expert teams conducted a total of 150 analyses for patients in the intervention group. All analyses were performed in the period of November 2013 to November 2014. On average, it took them 14.6 min (SD = 7.98) to complete a medication review, ranging from 2.1 to 42.7 min.

On average, the cases consisted of 13.4 (SD = 5.48) diagnosed diseases to which the users assigned 8.0 (SD = 4.66) medications. These medications comprised both ones patients already used, and new ones that the users prescribed.

### Clickstream

Clickstream analysis showed that the average number of recommendations that users followed was 1.01, which amounted to 16.16 % of the total of generated recommendations. Table 3 summarizes users’ responses to the recommendations the system generated.

**Table 3:** Summary of users’ responses to generated recommendations

	Team 1	Team 2	Team 3	Total
Average number of followed advices	8.80 % (M = 0.48, SD = 1.05)	13.69 % (M = 0.92, SD = 0.90)	25.00 % (M = 1.64, SD = 1.54)	16.16 % (M = 1.01, SD = 1.28)
Average number of rejected advices	47.62 % (M = 2.60, SD = 2.12)	28.27 % (M = 1.90, SD = 1.30)	20.43 % (M = 1.34, SD = 1.56)	31.20 % (M = 1.95, SD = 1.76)
Average number of ignored advices	43.58 % (M = 2.38, SD = 2.84)	59,04 % (M = 3.90, SD = 2.97)	54,57 % (M = 3.58, SD = 3.65)	52.64 % (M = 3.29, SD = 3.22)
	100 %	100 %	100 %	100 %

The first step, in which users were tasked with manually assigning medications to relevant diseases, was responsible for 46 % (6.0 min) of the time they spent on a medication review. Users spent 41 % (5.3 min) of the time on the second step, concerning underprescribing. The other phases took less time, with the final step being accountable for 1.8 % (0.3 min) of the time. Table 4 summarizes users’ time allocation during the medication review task.

**Table 4:** Summary of users’ time allocation per step

	Team 1	Team 2	Team 3	Total
Average time spent on medication assignment	44.47 % (M = 4.55, SD = 3.56)	53.00 % (M = 7.25, SD = 5.75)	40.74 % (M = 6.05, SD = 4.56)	46.16 % (M = 5.95, SD = 4.81)
Average time spent on underprescribing	43.79 % (M = 4.48, SD = 3.56)	36.04 % (M = 4.93, SD = 3.48)	44.44 % (M = 6.60, SD = 5.23)	41.43 % (M = 5.34, SD = 4.24)
Average time spent on overtreatment	5.47 % (M = 0.56, SD = 0.91)	4.24 % (M = 0.58, SD = 0.81)	5.72 % (M = 0.85, SD = 1.56)	5.20 % (M = 0.67, SD = 1.14)
Average time spent on clinical interactions	4.30 % (M = 0.44, SD = 0.93)	5.53 % (M = 0.77, SD = 1.32)	5.86 % (M = 0.87, SD = 1.70)	5.43 % (M = 0.70, SD = 1.35)
Average time spent on dosage adjustment	1.95 % (M = 0.20, SD = 0.46)	1.10 % (M = 0.15, SD = 0.22)	2.23 % (M = 0.48, SD = 2.07)	1.78 % (M = 0.23, SD = 1.23)
	100 %	100 %	100 %	100 %

### Efficiency

**Fig. 1:**
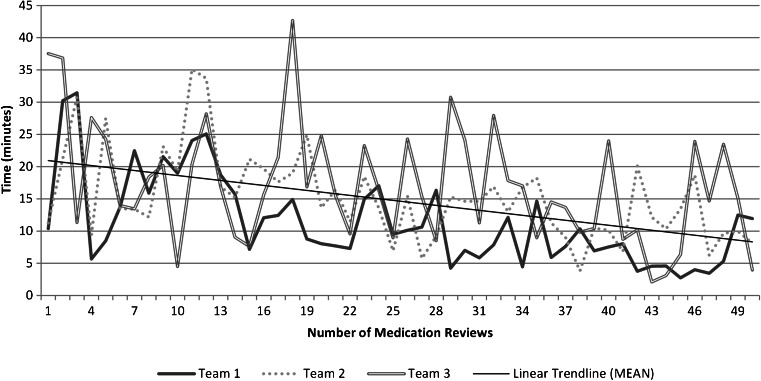
Average time spent per medication review over time

Figure 1 shows a decreasing trend in time spent on medication reviews over time. A fixed effects test on the linear mixed model showed a significant difference in the time required to conduct medication reviews over time; F(31.145) = 14.043, *p* < .001. The average time in minutes required to conduct medication reviews up to the first quartile was M = 20.42 (SD = 9.00), while the time from the third quartile up was M = 9.81 (SD = 6.13).

## Discussion

### Efficiency improvement

In this paper, the results of a longitudinal study on the effects of experience on physicians’ and pharmacists’ efficiency of performing decision-supported medication reviews were reported. In line with the authors’ hypothesis, the amount of time users needed to perform similar tasks decreased significantly as they gained experience.

Earlier validations of the STRIP method showed significant increases in effectiveness over usual care approaches, but decreases in efficiency [9, 19]. In the latest validation of the method embedded in a decision support system, the authors hypothesized that “experiments in which more gradual changes to the method are applied may result in improvements in both effectiveness and efficiency” [9]. This assumption was in line with the view proposed by Nilsson & Følstad of effectiveness and efficiency as conflicting requirements [28]. The results of this study confirm the hypothesis that users’ familiarity with an application and their experience with performing similar tasks leads to increases in efficiency.

Limited research has been done into the effects of temporality on usability in general, and efficiency in particular. Mendoza and Novick reported, though, that frustration levels of participants using unfamiliar software decreased over time, leading them to remark that “factors such as features being hard to find and operators committing slips and mistakes really are the principal causes of severe frustration” [4]. They report that with increased familiarity these frustrations decrease. It can be reasonably expected that, in a similar fashion, time spent on repetitive tasks decreases.

In fields related to usability, temporality has been recognized as being influential in shaping people’s motivations towards using software. In a user experience study, temporality appeared to be a determinant in the changing motivations of people who use software for a prolonged period of time: “prolonged experiences became increasingly more tied to aspects reflecting how the product becomes meaningful in one’s life” [5]. Experience has been shown to be a determinant in users’ attitudes towards acceptance of technology [2, 3, 7].

### Practical implications in STRIPA

The clickstream analysis pinpointed which steps of the STRIP process were least efficient and could be improved in the software application. In an attempt to decrease the workload of the first, medication assignment, step, knowledge discovery methods were employed. Based on historical data, the application was improved to automatically assign medications to appropriate diseases. Additionally, medication menus were redesigned to allow for quicker selection of probable choices. Clinical analysis of the recommendations that were most often rejected or ignored by users led to improvements in the clinical rule base which powers the decision support system.

## Conclusion

This paper reports the results of a longitudinal study on the effects of experience on physicians’ and pharmacists’ efficiency of performing decision-supported medication reviews. Corresponding with the authors’ hypothesis, the amount of time users needed to perform similar tasks decreased significantly as they gained experience.

### Limitations

Limitations of this study include the limited number of participants and the fact that the analyses were performed unsupervised. The participants were recruited as a convenience sample and are not representative of the population of clinical decision support systems users.

The effectiveness of the medication reviews that the users performed was not measured. Further research should reveal any changes in effectiveness over time, and if this is in any way correlated with the measured improvement in efficiency.
